# Rice Flour and Bran Enriched with Blueberry Polyphenols Increases Storage Stability and Decreases Arsenic Content in Bran

**DOI:** 10.3390/foods8070276

**Published:** 2019-07-23

**Authors:** Stephen M. Boue, Kim Daigle, John C. Beaulieu, Mark Heiman

**Affiliations:** 1 U.S. Department of Agriculture, Agricultural Research Service, Southern Regional Research Center, New Orleans, LA 70179, USA; 2 Microbiome Therapeutics, 11001 120th Ave, Broomfield, CO 80021, USA

**Keywords:** rice, flour, bran, blueberry, juice, pomace, polyphenols, anthocyanins, arsenic

## Abstract

A low-cost method utilizing rice co-products to concentrate and stabilize blueberry polyphenols was developed that decreased the arsenic (As) content in rice bran. After concentration at 10 g/L, brown rice flour displayed a higher total anthocyanin content in both blueberry juice (2.7 mg/g) and pomace extract (2.6 mg/g) when compared to white rice flour. Defatted rice bran enriched with blueberry juice (10 g/L) had the highest concentration of polyphenols (16.0 mg/g), and defatted bran enriched with pomace extract had the highest concentration of anthocyanins (5.32 mg/g). Enriched rice flour and bran contained higher levels of anthocyanins when using pomace extracts. Polyphenols and anthocyanins were found to be highly stable at 37 °C in rice flour and bran samples combined with pomace extract. Polyphenol enrichment also produced lower total and inorganic arsenic (i-As) levels in defatted rice bran. Inorganic arsenic (i-As) concentrations in defatted rice bran enriched with blueberry juice and pomace extracts were reduced by 20.5% and 51.6%, respectively. Overall, rice flour and bran that are enriched with polyphenols and anthocyanins from blueberry pomace extracts are shelf and color stable, had low sugar content, and represent unique health-promoting food ingredients.

## 1. Introduction

Rice is one of the most important grains that is used in all parts of world. Large amounts of rice are produced annually, including milled rice co-products such as broken kernels, rice flour, and rice bran. These underutilized co-products can serve as sources of value-added food ingredients. Rice flour contains more than 90% rice starch and other components including proteins, lipids, carbohydrates, and small amounts of minerals and vitamins. Rice flour also has the advantage of being gluten free and has become highly desirable because of the claims of gluten intolerance in the population [1]. Rice bran, a byproduct of rice milling, contains protein, lipids, dietary fibers, vitamins, minerals, vitamins, and phenolic compounds that have antioxidant activities [2,3,4]. The protein content of rice bran is between 14–20% and is often used as a source of food protein [5]. Rice bran is also undervalued, with a recent market value for a ton of rice bran at $95.00–$140.00. 

Although rice bran contains many health-promoting components, it also contains high levels of the carcinogen inorganic arsenic (i-As) [6,7]. Arsenic accumulates in the outer bran layer of rice and rice bran can contain up to between 10–20-fold higher levels of As [6]. Rice is typically grown using flooded rice paddies under anaerobic conditions where As from the soil is mobilized into the water and absorbed into the plant [7]. Much research has been devoted to developing methods to reduce arsenic accumulation in rice using different preharvest techniques [8,9]. Additionally, several postharvest methods have been developed to reduce As in rice during cooking [10,11]. These studies show that methods have been developed to better understand As in rice and rice bran, but further research is warranted when producing value-added products with rice bran.

Blueberries are a rich source of polyphenols such as anthocyanins, proanthocyanidins, polyphenol acids, and flavonols [12,13]. During blueberry juice processing, the leftover pomace is often discarded as waste. However, the blueberry pomace is a rich source of polyphenols and anthocyanins [14]. Blueberries have many health benefits that have been discovered. A blueberry-enriched diet was shown to improve insulin sensitivity [15] and lower the risk of Type 2 diabetes [16]. One problem with blueberries and blueberry juice is the high sugar content, which consists mainly of glucose and fructose. Studies have shown that blueberry powders that have their sugars and lipids removed are more effective at decreasing body weight gain when compared to whole blueberry powders [17,18]. Additionally, blueberry anthocyanins can be rapidly degraded with exposure to light, heat, and oxygen, and anthocyanins are known for their susceptibility to pH change [19].

Recently, research has shown the natural fortification of foods by the efficient sorption of fruit polyphenols (blueberry and cranberry juice) to soybean flour [20], and the use of defatted rice bran was used as a bioadsorbent for tea catechins [21]. Other research has shown hypoglycemic activity using a grape juice polyphenol–soybean flour complex [22]. Adding to the antidiabetic activity of the polyphenol-enriched food complexes is the added benefit of reducing the sugar content by binding only the polyphenol components of the sugar-rich fruit juices. This research focused on the ability of protein-rich soybean and other protein-rich foods as a binding matrix for juice polyphenols [20,22,23,24,25].

Although soybean flour and proteins have been shown to be a good matrix for polyphenols, little research has explored the use of rice co-products for binding polyphenols. The purpose of this study was to develop rice functional food ingredients utilizing rice flour and bran that would be low in sugar content, shelf stable, and a concentrated source of natural blueberry polyphenols. This study utilized rice co-products, particularly rice bran, and blueberry pomace, which are typically considered waste products. Additionally, our study compared the use of both blueberry juice and extracted pomace to produce polyphenol-enriched rice bran and flour food matrices. We examined the color and polyphenol stability during the storage of both food ingredients, as well as the arsenic content of rice bran before and after enrichment with polyphenols.

## 2. Materials and Methods

### 2.1. Materials

Folin–Ciocalteu reagent, cyanidin-3-glucoside, (+)-catechin, gallic acid, acetonitrile, and ethanol were obtained from Sigma-Aldrich (St. Louis, MO, USA). Rice (Cypress variety) was provided by the LSU AgCenter Rice Research Station. Rice hulls were removed from paddy rice using a Satake Paddy Husker (Type HU, Satake Engineering Co., Hiroshima, Japan). After removing the hulls from the rough rice, whole grain rice was milled using a Satake Grain Testing Mill (model TM 05, Satake Engineering Co., Hiroshima, Japan); then, the full-fat bran was isolated after sieving through a 590-μm mesh sieve (E. H. Sargent & Co., Chicago, IL, USA). Defatted bran was obtained using the following method: full-fat bran (120 g) was extracted with hexane (2×) at a 1:5 bran to solvent ratio with shaking at 200 rpm for 1 h at 25 °C, and then centrifuged (7000× *g*, 4 °C, 15 min). Brown flour (rice after dehulling) and white flour (rice after bran removal) were obtained using an Udy mill (Udy Corporation, Fort Collins, CO, USA) with a 0.5-mm mesh sieve. The protein content of rice samples was analyzed using a Leco nitrogen analyzer (St. Joseph, MI, USA): brown flour (8.0%), white flour (7.7%), defatted bran (14.6%), and full-fat bran (13.5%).

Frozen Tifblue blueberries (*Vaccinium ashei*) were purchased from Blue River Farms, LLC (Mt. Olive, MS, USA). A juicing machine (Braun Model 4200) was used for juice and pomace production. Juice was centrifuged (1500× *g* for 10 min) and vacuum filtered (Whatman 4). Pomace was vacuum filtered to remove excess juice, frozen, and lyophilized. Lyophilized blueberry pomace was milled using a Glen Mills (Clifton, NJ, USA) MHM4 hammer mill with a 0.5-mm sieve. Blueberry pomace was extracted with water and citric acid buffer. A citric acid (5 g) and sodium citrate (3 g) buffer with 80 mL of water (pH = 3.5) was utilized for pomace extractions at 4 °C, 25 °C, and 60 °C. 

### 2.2. Preparation of Rice Blueberry Complexes 

Rice–blueberry complexes were produced using the method of Roopchand et al. [20] with minor modifications. Briefly, rice flour and bran were mixed with 10 mL of blueberry juice or pomace extracts with stirring for 10 min at 23 °C. Amounts of rice flour and bran were varied with 0.1 g in the 10 g/L complex, 0.5 g in the 50 g/L complex, and 1.0 g in the 100 g/L complex. Samples were centrifuged for 10 min at 11,750× *g*, and the supernatants were decanted. Polyphenol-enriched flour and bran samples were freeze-dried using a Virtis Genesis (25L Genesis SQ Super XL-70, SP Industries, Warminster, PA, USA) with the shelf temperature initially set to −30 °C for 72 h. Triplicate samples were prepared for each sample. 

### 2.3. Polyphenol and Anthocyanin Analysis 

Polyphenol-enriched flour and bran samples (0.1 g) were extracted with 2 mL of 1% trifluoroacetic acid in 80% methanol. Samples were sonicated for 1 h, centrifuged at 11,750× *g* for 10 min, and filtered (2 µm). The Folin–Ciocalteu reagent method was used to determine the total polyphenol content in the rice bran extracts. The total polyphenol content was expressed as gallic acid equivalents (GAE) in milligrams per gram of sample using a standard curve. Monomeric anthocyanin content was determined using the pH differential method. Each extract was appropriately diluted using pH 1.0 and 4.5 buffers. Absorbance was read on 1-cm path-length disposable cuvettes at 510 nm and 700 nm. Results are expressed as cyanidin-3-glucoside (C3G) equivalents. 

### 2.4. Storage Stability of Polyphenols and Anthocyanins 

Multiple 1 g samples of freeze-dried blueberry-polyphenol rice flour and bran samples (50 g/L concentration of rice/juice or pomace extract) were placed into 10-mL screw-cap vials, divided into three sets, and incubated at 37 °C. Samples were incubated in large glass desiccators, sealed, and placed in a Lindberg Blue M oven (Model 1490, Riverside, MI, USA). Additionally, blueberry pomace and juice were lyophilized. Samples were removed at the indicated weeks and analyzed for total polyphenol and total anthocyanin contents.

### 2.5. Colorimetric Parameters 

The tristimulus Hunter L*, a*, and b* values of brown rice flour and defatted rice bran enriched with juice and pomace extracts were measured by reflectance using a colorimeter (CR-400; Konica-Minolta, Tokyo, Japan). Five readings were made separately from three different spots at sample cases (transparent mini sample dishes made of polypropylene) containing rice–blueberry complexes, and the mean values were reported. Color functions such as total color difference (∆E), hue angle (h), and chroma values (C*) were calculated from the Hunter L*, a*, and b* values.

### 2.6. Glucose, Fructose, and Sucrose Analysis 

Rice–blueberry samples (100 mg) were homogenized in 1 mL of water and thoroughly mixed by vortexing for 30 min. Samples were centrifuged at 1050× *g* for 10 min. The supernatants (50 µL) were assayed using a Megazyme K-SUFRG kit (Megazyme, Wicklow, Ireland) according to directions supplied by the manufacturer. All the spectrophotometric measurements were performed on a Shimadzu UV-1800 spectrophotometer (λ = 340 nm, Shimadzu Inc., Kyoto, Japan). 

### 2.7. Arsenic Analysis 

For total arsenic, bran samples (1 g) were mixed with 5 mL of HNO_3_, 2 mL of 25% HCl, and 1.5 mL of H_2_O, and digested in a Milestone Ultrawave microwave system pressurized to 40 bar, slowly ramping to 260 °C and holding for 25 min (pressure in the chamber during digestion will reach 130 bar), cooled, and diluted to 15-mL volume with deionized water. Total As in the digests was measured using hydride generation. Using a mixing block, the pre-reduced solutions are combined with 0.5% sodium borohydride in 0.05% sodium hydroxide in order to analyze for total As using a flow injection hydride generation system with inductively coupled plasma-atomic emission spectroscopy (ICP-AES; Perkin Elmer Optima 4300DV ICP-OES, Waltham, MA, USA). The method for inorganic arsenic developed by Chaney, Green, and Lehotay was used [26]. A sample size of 0.7 g of bran was weighed out and mixed with 10 mL of 0.28 M HNO_3_; then, it was digested in a preheated DigiPrep Hotblock at 95 °C for 90 min, filtered, and brought to a 20-mL final volume with 0.28 M of HNO_3_. The i-As in the extractions was measured using hydride generation. The i-As was analyzed using inductively coupled plasma atomic emission spectroscopy (ICP-AES). 

### 2.8. Statistical Analysis 

Experimental data were subjected to one-way analysis of variance (ANOVA) to determine significant differences. Duncan’s multiple range test at *p* < 0.05 were carried out using XLStat for Excel version 2018.5 (Addinsoft Software, Inc., Long Island City, NY, USA).

## 3. Results and Discussion

### 3.1. Blueberry Juice Production and Pomace Extractions

The pressing and grinding of blueberries produced juice (pH = 3.1) without any enzymatic treatment and pomace. Previous research on the enrichment of soybean flour with grape polyphenols determined that the protein component was binding with polyphenols [22] and the amount of polyphenols or anthocyanins sorbed to the flour remained optimal at pH 3.5. In this study (data not shown), results indicated that this same pH (pH = 3.5) was optimal for polyphenols and anthocyanins binding with rice bran and flour. In order to assist complex formation between rice proteins and blueberry polyphenols, a citric acid buffer at pH 3.5 was chosen for the extraction of blueberry pomace. Citric acid has been shown to increase the extraction of polyphenols, particularly anthocyanins, from plant samples [27,28]. Acidic solvents have also been shown to assist with the extraction of anthocyanins and other compounds found in blueberry samples [28]. A comparison for the extraction of polyphenols and anthocyanins in pomace using both water and citric acid buffer is reported in Table S1. Initial experiments utilized water and citric acid buffer at a 10:1 solvent:pomace ratio, 4 °C, 25 °C, and 60 °C temperatures, and stirring for 1 h. Significantly higher levels of polyphenols (9.20 mg/g) and anthocyanins (4.21 mg/g) were extracted at 60 °C using the citric acid buffer. Therefore, further experiments varied the solvent:pomace ratio with citric acid buffer and 60 °C temperatures. Further increases in both polyphenol content and anthocyanin levels were reported after increasing the extraction time to 3 h. The highest levels of polyphenols (18.6 mg/g) and anthocyanins (9.11 mg/g) were determined with a 20:1 solvent:pomace ratio and a 3-h extraction time, and these conditions were used for pomace extractions when combined with rice co-products throughout this study. 

### 3.2. Polyphenol and Anthocyanin Content of Blueberry-Enriched Rice Flour and Bran

**Figure 1:**
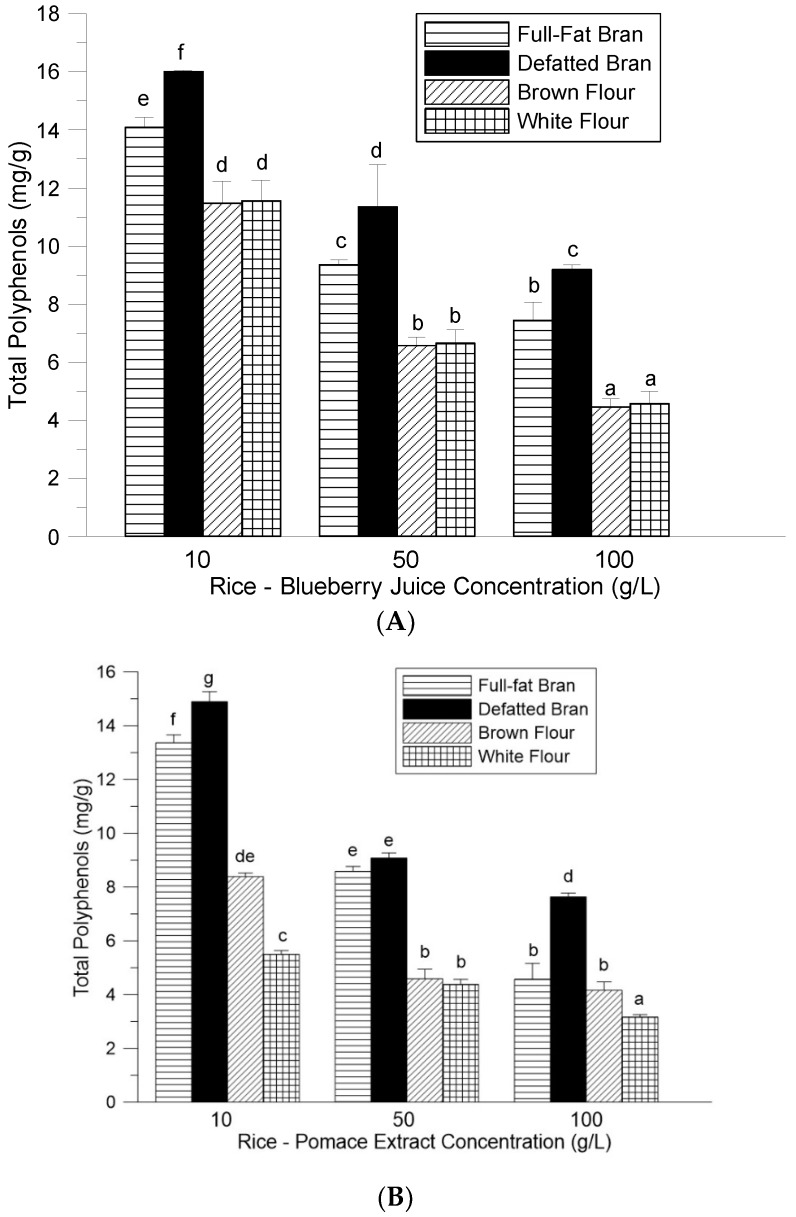
Total polyphenol content (mg GAE/g) of rice bran and flour with (**A**) blueberry juice and (**B**) blueberry pomace. Each bar represents the mean ± SD of data combined from three independent experiments. Means with different letters (a–g) are significantly (*p* < 0.05, Duncan multiple range test) different. GAE: gallic acid equivalents; SD: standard deviation.

The ability of rice flour and bran to bind blueberry polyphenols and anthocyanins is shown in Figure 1A,B. Three different concentrations (10–100 g rice/L blueberry juice or pomace extract) were compared, and the resulting dry complexes were analyzed. Defatted rice bran contained the highest levels of polyphenols of the rice co-products tested at each of the three concentrations of added juice, with the highest polyphenol content produced using 10 g/L (16.0 mg/g). Using blueberry pomace produced similar binding activity, with the defatted bran having the highest polyphenol content of 14.8 mg/g at 10 g/L and full-fat bran having lower polyphenol content of 13.4 mg/g at 10 g/L. Full-fat bran also showed an ability to bind juice polyphenols with the highest polyphenol content of 14.0 mg/g at 10 g/L. Defatted rice bran has a protein content of 14.6%, which was higher than that of the full-fat bran at 13.5%. This higher protein content is an explanation for the higher polyphenol content found in the defatted bran samples. No statistical differences were found when comparing brown rice flour with white flour when adding blueberry juice to these rice matrices. However, for the pomace extract and flour complexes, the polyphenol content of the brown rice flour was higher at both the 10 g/L and 100 g/L concentrations, respectively. There was a small difference in protein content between brown rice flour at 8% and polished white rice flour at 7.7% that may account for the ability of brown rice to bind polyphenols at higher amounts compared to white flour. The blueberry juice in our study contained a total polyphenol content of 1.29 mg/mL (10 mL of juice would contain 12.9 mg of GAE), which was higher than the amount of total polyphenol content obtained in the pomace extract at 18.6 mg/g (10 mL of pomace extract would contain 9.3 mg of GAE). The higher polyphenol content of the blueberry juice in our study accounts for the observed higher polyphenol content of rice flour and bran enriched with juice when compared to rice samples enriched with pomace extract. 

**Figure 2:**
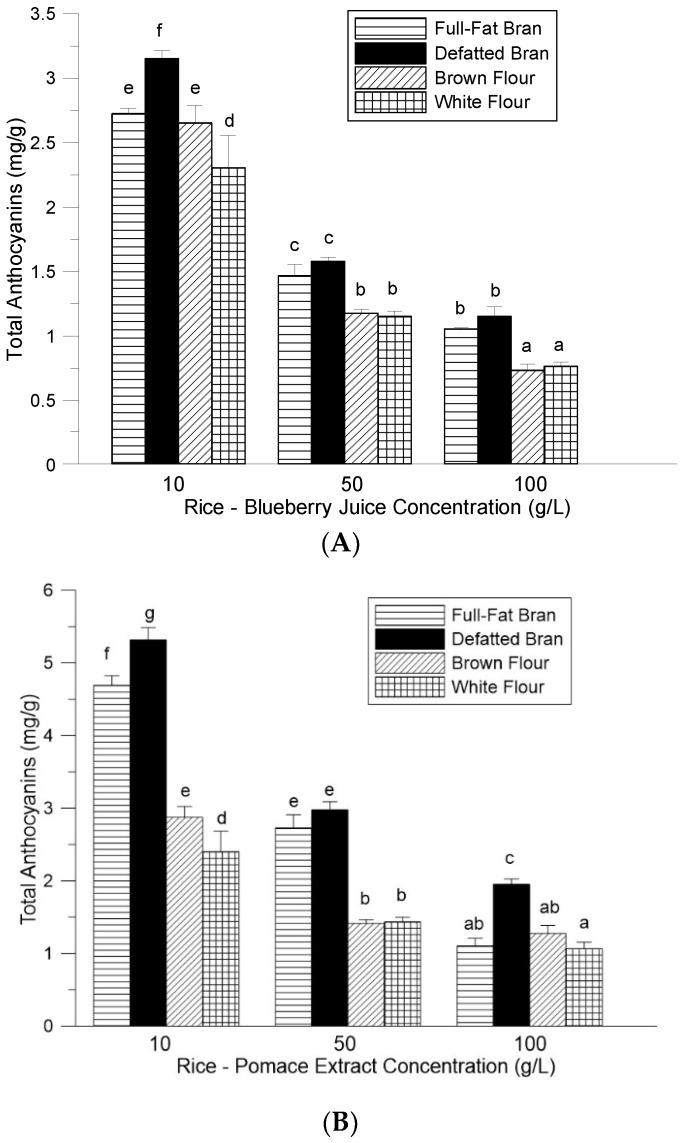
Total anthocyanin content (mg C3G/g) of rice bran and flour with (**A**) blueberry juice and (**B**) blueberry pomace. Each bar represents the mean ± SD of data combined from three independent experiments. Means with different letters (a–g) are significantly (*p* < 0.05, Duncan multiple range test) different. C3G: cyanidin-3-glucoside; SD: standard deviation.

Rice flour and bran displayed similar abilities to bind anthocyanins. The initial total anthocyanin contents of both rice bran and flour were determined after the addition of blueberry juice (Figure 2A) and pomace extract (Figure 2B). The anthocyanin contents of the defatted bran samples at 10 g/L juice concentration and 10 g/L and 100 g/L pomace extract concentrations were significantly higher than that of the full-fat bran at these concentrations. However, all of the other sample concentrations were not statistically different. Overall, the anthocyanin contents were higher using the pomace extracts, with the highest level detected for the defatted rice bran at 10 g/L of 5.32 mg/g (C3G) compared to 3.15 mg/g (C3G) for the defatted rice bran at 10 g/L using juice. Also, the anthocyanin content of the brown rice flour enriched with pomace extract was significantly higher than the white rice flour sample at only the 10 g/L concentration. The brown rice flour and white flour samples enriched with juice were not statistically different. The blueberry juice in this study contained a total anthocyanin content of 0.275 mg/mL (10 mL of juice would contain 2.75 mg of C3G), which was lower than the total anthocyanin content detected in the pomace extract at 9.11 mg/g GAE (10 mL of pomace extract would contain 4.55 mg of C3G). The higher anthocyanin content of the pomace extract used in this study explains the overall observed higher anthocyanin contents of rice samples enriched with pomace extract when compared to rice samples enriched with juice. 

### 3.3. Changes in Sugar Composition

One aspect of enriching rice bran and flour with blueberry components is the ability of the rice samples to bind polyphenols rather than the sugar components present in blueberry juice and pomace extracts. Table 1 shows the glucose, fructose, and sucrose contents of dried blueberry juice, pomace, and brown flour and defatted bran enriched with juice and pomace extract. Dried blueberry juice had a high content of glucose and sucrose with a total sugar content of 715 mg/g. Pomace had lower levels of glucose and fructose with a total sugar content of 131 mg/g. Defatted bran and brown flour combined with juice had 227 mg/g and 163 mg/g total sugar content, respectively. However, much lower total sugar contents were observed with defatted bran and brown flour enriched with pomace extracts at 28.6 mg/g and 15.3 mg/g, respectively. 

Much research is currently ongoing to develop low-sugar food ingredients that contain health-promoting polyphenols, particularly in the area of low-sugar food ingredients. Blueberries and other fruits contain high levels of glucose and fructose, which may counter the antidiabetic potential of anthocyanins and other polyphenols. Roopchand et al. [20] showed that the enrichment of defatted soybean flour with blueberry juice caused polyphenols (anthocyanins) to bind with the food matrix (proteins), and no glucose or other sugars would be retained. Our research indicates that low levels of glucose and fructose from blueberry juice are retained in the defatted rice bran and flour. In contrast, significantly lower levels of these sugars are retained when using the pomace extract. 

### 3.4. Changes in Polyphenols and Anthocyanins during Storage

**Figure 3:**
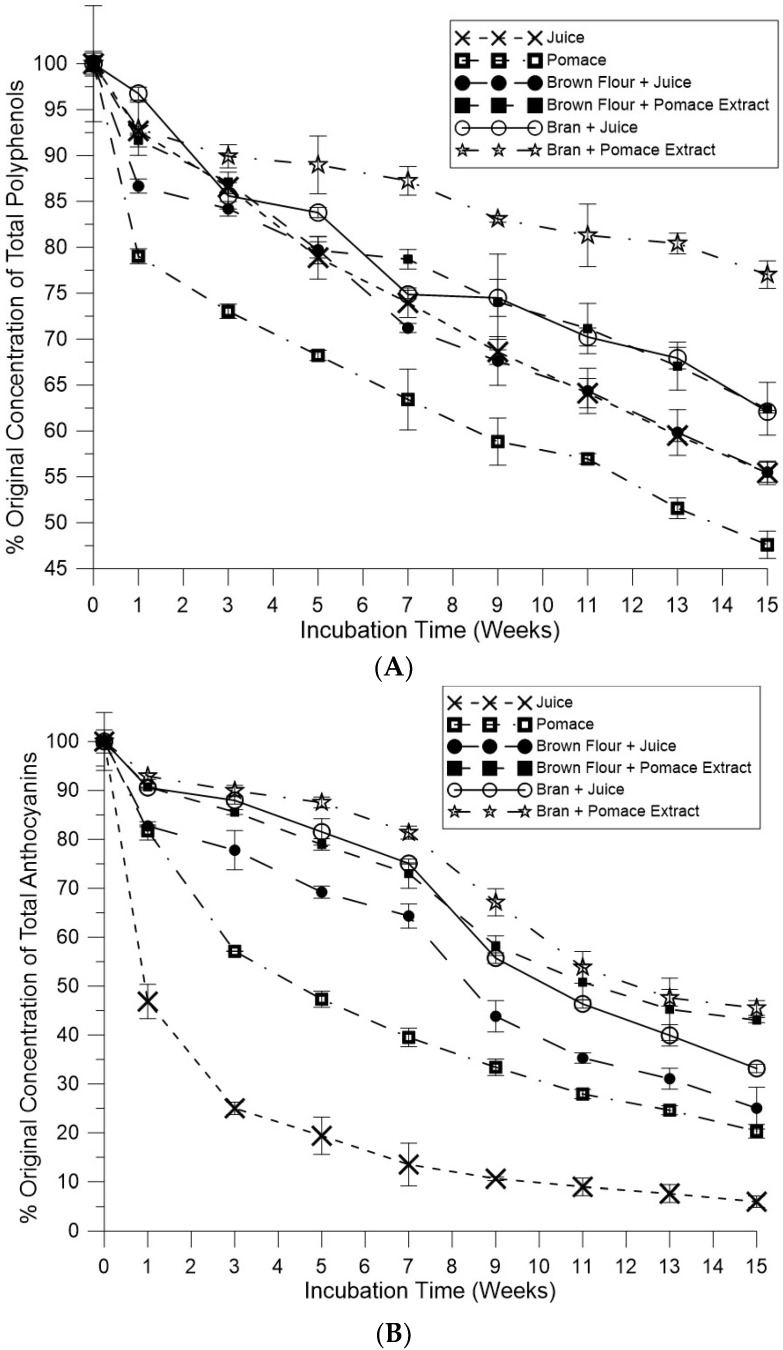
Storage stability at 37 °C of blueberry polyphenols and anthocyanins bound to defatted rice bran and brown rice flour (50 g/L concentration of rice/juice or pomace extract). (**A**) Concentration of polyphenols extracted from blueberry polyphenol-enriched rice bran and flour after the indicated number of weeks; (**B**) Concentration of anthocyanins extracted from blueberry polyphenol-enriched rice bran and flour after the indicated number of weeks.

The effects of storage on polyphenols and anthocyanins were determined for brown rice flour and defatted rice bran complexes. The change in the total polyphenol contents of brown rice flour and defatted bran blueberry complexes after storage at 37 °C is shown in Figure 3A. Dried blueberry pomace demonstrated the lowest stability over the 15-week storage period with decreased polyphenol content falling to 47.6% of the original polyphenol concentration by week 15. Lyophilized blueberry juice decreased to 55.4% of its original polyphenol concentration during storage. The bran combined with citric acid blueberry pomace decreased the least, to only 77.0% (loss of 23%) at week 15. Overall, the bran and brown flour samples, when combined with the blueberry citric acid pomace extracts, decreased less when compared to the bran and brown flour samples combined with blueberry juice.

The change in total anthocyanin content of brown rice flour and defatted bran blueberry complexes after 37 °C storage is shown in Figure 3B. The dried blueberry juice sample displayed the largest decrease in anthocyanin content, to just 6% after the 15-week storage period. There were higher levels of anthocyanins in the pomace sample (20.3% original concentration) compared to dried juice (6% original concentration). The anthocyanins from brown rice flour and bran combined with blueberry pomace extract decreased the least. The rice bran + pomace extract decreased to 45.5% of the original total anthocyanin content after 15 weeks of storage, followed by the brown rice flour + pomace extract at 43.0%. The rice bran and brown rice flour samples combined with blueberry juice decreased to 33.2% and 25.1% of the original anthocyanin content during storage. One possible reason for the difference in stabilities is the addition of citric acid used in the pomace extract, which has a stabilization effect on polyphenols [29]. 

A trend observed in both Figure 3A,B was that defatted bran samples, treated with blueberry juice and pomace extracts, had less decreases in polyphenol and anthocyanin contents compared to brown rice flour. One possible explanation for this is the higher protein content (13.5%) of the rice bran sample compared to the1 only 8% protein content of the brown rice flour sample. Previous research has shown that polyphenols have the ability to bind with plant proteins, which may enable a higher degree of storage stability [20,22,23,24,25,30]. 

### 3.5. Changes in Color Parameters during Storage

Color parameters of the defatted rice bran and brown rice flour enriched with blueberry polyphenols from juice and pomace extracts, at the beginning and the end of storage (37 °C), are shown in Table 2. After 105 days of storage, all the samples displayed a significant increase in lightness (L*). Brown rice flour and defatted bran when enriched with juice showed a larger increase in lightness—42.1% and 46.2%, respectively—when compared to brown rice flour and defatted bran combined with the pomace extract, which were 32.1% and 29.4%, respectively. An increase in the L* values in model systems based on purple corn and red cabbage has been interpreted as an indication of discoloration due to anthocyanin degradation [31,32]. The color intensity (C*) of all the rice bran and flour-enriched samples increased significantly after storage, and thus they appeared duller. The hue angle value is the attribute of color that is perceived or the color tone (blue, red, green, yellow, etc.). In Table 2, the rice flour and bran samples enriched with juice showed larger hue angle shifts when compared to the rice samples enriched with pomace extracts. Differences in hue angle have been attributed to differences in anthocyanin and polyphenol composition [33]. In general, this larger hue angle change is associated with the development of products of anthocyanin degradation. 

For all the samples, there was a significant effect of the source of blueberry polyphenols on the total color difference (ΔE) between the beginning and the end of storage. The total color difference calculated from changes of the three components L*, a*, and b*, were all higher than one (threshold assumed as a basis for a color difference detected by the human eye). The total color difference was higher in the brown rice flour and defatted bran samples enriched with blueberry juice at 13.3 and 14.7, respectively. The smallest changes in ΔE was found in brown rice flour (11.1) and full-fat bran (11.5) samples enriched with pomace extract. The use of the pomace extract inhibited the color changes observed with use of juice, and indicates the delayed degradation of anthocyanins and polyphenols in the samples during storage (shown in Figure 3A,B). 

### 3.6. Total and Inorganic Arsenic Content in Rice Bran 

The effect of polyphenol enrichment on the total arsenic concentrations of defatted rice bran is shown in Table 3. The control defatted rice bran (no polyphenolic enrichment) had 0.847 ppm of total As; however, both the blueberry juice-enriched bran and blueberry pomace-enriched bran samples had significantly lower levels of total arsenic. The enrichment of defatted bran utilizing blueberry juice lowered the total As levels by 21.1% to 0.668 ppm, and blueberry pomace extract lowered the total arsenic levels by 47.5% to 0.445 ppm. The control defatted rice bran had 0.610 ppm i-As (Table 3), which was also reduced by 20.5% to 0.485 ppm i-As in the rice bran–blueberry juice treatment, and by 51.6% to 0.295 ppm i-As in the rice bran–blueberry pomace extract treatment. 

Much research has demonstrated that rice accumulates arsenic in the bran layer at high levels for several reasons, including the use of flooded fields and anaerobic conditions during plant growth [8]. Preharvest methods to reduce arsenic levels in rice involve modified rice farming methods [8,9] and the development of rice varieties that absorb less arsenic from the soil and water [34]. Research has shown that rinsing rice before cooking can reduce arsenic by up to 29% [35], and decanting excess water after high-volume cooking can effectively decrease both total and i-As in rice [36]. This research on the reduction of arsenic in whole rice demonstrates a potential method that can also be applied to reduce arsenic in rice bran, particularly methods using excess water with the removal of some of the water to remove arsenic. This current study with rice bran utilized a method to enrich the polyphenol content of the bran by placing the bran sample in excess blueberry juice or pomace extract. After a 10-min incubation period, the rice bran was centrifuged, and the juice or pomace extract was decanted. This method significantly reduced both the total and i-As contents of the rice bran. 

Arsenic in rice can exist in several forms, but is predominately arsenite (AsIII), arsenate (AsV), and dimethylarsinic acid (DMA) [37]. Water appears to have the ability to remove i-As when rice is cooked in excess water. In a study by Raab et al. [36], the average pH of water that was used in excess to cook rice was 6.2. One hypothesis for lowering i-As preferentially is that aresenite is uncharged in water (pKa1 = 9.22), and is more mobile and more likely to migrate from the rice grain than arsenate (pKa1 = 2.20) or DMA (pKa1 = 6.1) [36]. Arsenite was almost exclusively observed in the excess cooking water, supporting this hypothesis. In our study, the control rice bran’s As composition was 71.8% i-As and 28.2% organic arsenic (o-As) (calculated by difference), the bran enriched with blueberry juice was comprised of 72.6% i-As and 27.4% o-As, and the bran enriched with the blueberry pomace extract was comprised of 66.3% i-As and 33.7% o-As. The pH plays an important role in determining the state (arsenite or arsenate) of arsenic. Although the pH values of the blueberry juice (pH = 3.1) and pomace extract (pH = 3.5) were similar, only the pomace extract was able to preferentially reduce the i-As composition below that of the control bran and bran–blueberry juice samples. The slightly higher pH value of the pomace extract may explain the higher reduction in i-As for this sample. Another unique aspect of the pomace extract used in this study was the addition of citric acid to the water for the extraction of polyphenols from pomace. Research has shown that citric acid can be a useful complexing agent (with Fe (II) present) in the removal of arsenic from water [38,39]; however, further testing is warranted to examine the ability of citric acid to aid in the removal of arsenic from rice bran (with and without added pomace polyphenols). Additionally, both the i-As content of rice bran after the addition of blueberry pomace extract (0.295 ppm) and the total As content (0.445 ppm) were still above the 0.200 ppm maximum level in polished rice recommended in 2014 by the Codex Alimentarious Commision’s Committee on Contaminants in Foods [40]. To reduce rice bran below this maximum level would require starting with a lower i-As content rice (bran), particularly for newer rice varieties that are being developed with lower As content [34]. 

## 4. Conclusions

Rice co-products were enriched with polyphenols and anthocyanins by utilizing both blueberry juice and pomace extracts. Brown and white rice flour displayed similar abilities to retain polyphenols; however, defatted rice bran contained higher concentrations of polyphenols and anthocyanins when compared to full-fat rice bran. We hypothesize that the higher protein content of the defatted rice bran enables a higher degree of protein–polyphenol complexation. Results show that polyphenols and anthocyanins were stable after storage in the rice bran and flour samples enriched with pomace extract. The pomace extract-enriched rice samples also displayed color stability after storage. Lastly, total and inorganic arsenic levels in defatted rice bran enriched with blueberry pomace extracts were reduced by 47.5% and 51.6%, respectively. Overall, rice co-products enriched with anthocyanins and polyphenols utilizing blueberry pomace extracts provide unique food ingredients that are shelf and color stable, contain low sugar content, and reduced the arsenic content of bran.

## Figures and Tables

**Table 1:** Sugar composition of rice co-products combined with blueberry juice and citric acid pomace extract (50 g/L concentration of rice/juice or pomace extract).

	Glucose (mg/g)	Fructose (mg/g)	Sucrose (mg/g)	Total (mg/g)
Blueberry Juice (Dried)	359 ± 1.2 ^f^	355 ± 1.7 ^f^	0.61 ± 0.07	715 ± 0.64 ^f^
Brown Flour + Juice	81.3 ± 0.49 ^d^	81.5 ± 0.49 ^d^	N/D	163 ± 0.71 ^d^
Defatted Bran + Juice	113 ± 0.24 ^e^	113 ± 1.2 ^e^	N/D	227 ± 1.0 ^e^
Pomace	65.4 ± 2.9 ^c^	65.2 ± 3.4 ^c^	N/D	131 ± 6.3 ^c^
Brown Flour + Pomace Extract	7.81 ± 0.21 ^a^	7.48 ± 0.28 ^a^	N/D	15.3 ± 0.49 ^a^
Defatted Bran + Pomace Extract	14.5 ± 0.53 ^b^	14.1 ± 0.24 ^b^	N/D	28.6 ± 0.28 ^b^

Values represent means ± SDs. N/D not detected. All means for glucose, fructose, and sucrose with different letters (a to f) within the same column are significantly (*p* < 0.05, Duncan multiple range test) different. SD: standard deviation.

**Table 2:** Changes in color parameters for brown flour and defatted rice bran combined with blueberry complexes during storage (50 g/L concentration of rice/juice or pomace extract).

Rice–Blueberry Enrichment	Days	Lightness (L*)	Chroma (C*)	Hue Angle (h)	Color Difference (ΔE)
Brown Flour + Juice	0	30.9 ± 0.1 ^a^	10.3 ± 0.1 ^a^	13.4 ± 0.4 ^d^	13.3 ± 0.8 ^b^
	105	43.9 ± 0.8 ^de^	13.4 ± 0.4 ^d^	9.01 ± 0.4 ^d^	
Brown Flour + Pomace Extract	0	32.7 ± 0.9 ^b^	11.8 ± 0.3 ^c^	356.9 ± 0.152 ^f^	11.1 ± 0.2 ^a^
	105	43.2 ± 0.1 ^cd^	15.5 ± 0.4 ^f^	357.8 ± 0.072 ^g^	
Defatted Bran + Juice	0	30.5 ± 0.6 ^a^	10.6 ± 0.1 ^a^	1.82 ± 0.1 ^a^	14.7 ± 0.4 ^c^
	105	44.6 ± 0.5 ^f^	14.0 ± 0.1 ^e^	14.1 ± 0.2 ^e^	
Defatted Bran + Pomace Extract	0	33.0 ± 0.9 ^b^	11.2 ± 0.4 ^b^	358.3 ± 0.3 ^h^	11.5 ± 0.4 ^a^
	105	42.7 ± 0.5 ^c^	17.5 ± 0.1 ^g^	2.6 ± 0.09 ^b^	

Values are mean ± SD (*n* = 3). Means with different letters (a to h) within the same column are significantly (*p* < 0.05, Duncan multiple range test) different. SD: standard deviation.

**Table 3:** Changes in (A) total arsenic and (B) inorganic arsenic (i-As) content in defatted rice bran before and after enrichment with blueberry juice and pomace extract (50 g/L concentration of rice/juice or pomace extract).

	Inorganic As (ppm)	Total As (ppm)
Rice Bran	0.610 ± 0.015 ^c^	0.847 ± 0.021 ^c^
Rice Bran + Juice	0.485 ± 0.017 ^b^	0.668 ± 0.018 ^b^
Rice Bran + Pomace Extract	0.295 ± 0.019 ^a^	0.445 ± 0.011 ^a^

Values are mean ± SD (*n* = 3). Means with different letters (a to c) within the same column are significantly (*p* < 0.05, Duncan multiple range test) different. SD: standard deviation.

## Supplementary Materials

Supplementary materials are available at https://www.mdpi.com/2304-8158/8/7/276/s1. Table S1: Total polyphenol content and anthocyanin content of blueberry pomace after extraction with water and citric acid buffer with varying extraction time, solvent to pomace ratio, and temperature. Means with different letters in each column (a-l) are significantly (*p* < 0.05) different. GAE: gallic acid equivalents.
