# Supplementary material for: Rice Flour and Bran Enriched with Blueberry Polyphenols Increases Storage Stability and Decreases Arsenic Content in Bran
Source: Foods. 2019 Jul 23;8(7):276. doi: 10.3390/foods8070276 (PMC6678838; doi:10.3390/foods8070276)
Supplement: Supplementary file 1 [file foods-08-00276-s001.pdf]

**Table S1**. Total polyphenolic and anthocyanin content of blueberry pomace after extraction with water and citric acid buffer.

| Solvent | Solvent: Pomace Ratio | Temperature (ºC) | Extraction Time (h) | Total Anthocyanins (mg/g) | Total Polyphenols (mg/g) |
|---|---|---|---|---|---|
| Water | 10:1 | 4 | 1 | 1.05 ± 0.21 a | 3.29 ± 0.02 a |
| | 10:1 | 25 | 1 | 1.32 ± 0.06 a | 4.15 ± 0.07 b |
| | 10:1 | 60 | 1 | 2.62 ± 0.05 b | 7.87 ± 0.16 e |
| Citric Acid Buffer | 10:1 | 4 | 1 | 2.86 ± 0.10 b | 5.44 ± 0.05 c |
| | 10:1 | 25 | 1 | 3.65 ± 0.11 c | 6.92 ± 0.08 d |
| | 10:1 | 60 | 1 | 4.21 ± 0.12 d | 9.20 ± 0.02 f |
| | 10:1 | 60 | 2 | 4.58 ± 0.13 e | 9.98 ± 0.06 g |
| | 10:1 | 60 | 3 | 5.38 ± 0.24 f | 10.7 ± 0.19 h |
| | 15:1 | 60 | 1 | 5.76 ± 0.06 g | 11.1 ± 0.14 i |
| | 15:1 | 60 | 2 | 6.63 ± 0.11 h | 12.9 ± 0.08 j |
| | 15:1 | 60 | 3 | 7.65 ± 0.13 j | 14.8 ± 0.31 k |
| | 20:1 | 60 | 1 | 7.24 ± 0.11 i | 13.2 ± 0.25 j |
| | 20:1 | 60 | 2 | 7.65 ± 0.41 j | 14.9 ± 0.05 k |
| | 20:1 | 60 | 3 | 9.11 ± 0.05 k | 18.6 ± 0.28 l |

Data are expressed at mean ± SD. Different letters in each column are statistically significant ($p < 0.05$)
